# Extractor for ESI quadrupole TOF tandem MS data enabled for high throughput batch processing

**DOI:** 10.1186/1471-2105-5-162

**Published:** 2004-10-26

**Authors:** Andreas M Boehm, Robert P Galvin, Albert Sickmann

**Affiliations:** 1 Protein Mass Spectrometry and Functional Proteomics Group, Rudolf-Virchow-Center for Experimental Biomedicine, Universitaet Wuerzburg, Versbacher Strasse 9, D-97078 Wuerzburg, Germany; 2 Applied Biosystems, Applera UK, Lingley House, 120 Birchwood Boulevard, Warrington, Cheshire, WA3 7QH, UK

## Abstract

**Background:**

Mass spectrometry based proteomics result in huge amounts of data that has to be processed in real time in order to efficiently feed identification algorithms and to easily integrate in automated environments. We present wiff2dta, a tool created to convert MS/MS data obtained using Applied Biosystem's QStar and QTrap 2000 and 4000 series.

**Results:**

Comparing the performance of wiff2dta with the standard tools, we find wiff2dta being the fastest solution for extracting spectrum data from ABIs raw file format. wiff2dta is at least 10% faster than the standard tools. It is also capable of batch processing and can be easily integrated in high throughput environments. The program is freely available via ,  and is also available from Applied Biosystems.

**Conclusions:**

wiff2dta offers the possibility to run as stand-alone application or within a batch process as command-line tool integrated in automation and high-throughput environments. It is more efficient than the state-of-the-art tools provided.

## Background

In tandem mass spectrometry proteins are identified by matching the measured fragment ion spectra derived from peptides with theoretical spectra calculated from known DNA or protein sequences, for example the NCBI sequence database [1]. Algorithms used for this purpose usually have their own input formats and are not able to read the proprietary binary file formats of the mass spectrometer manufacturers. Nevertheless, they are able to read a common format, the DTA format introduced by the Sequest™ algorithm [2]. Thus, needs exist for converting mass spectra into this common format in order to feed the different identification algorithms such as Sequest™ or Mascot™ [3]. The conversion must be accomplished efficiently, requiring as few user interaction as possible. Integrated in high-throughput environments, mass data processing must be realized.

**Figure 1:**
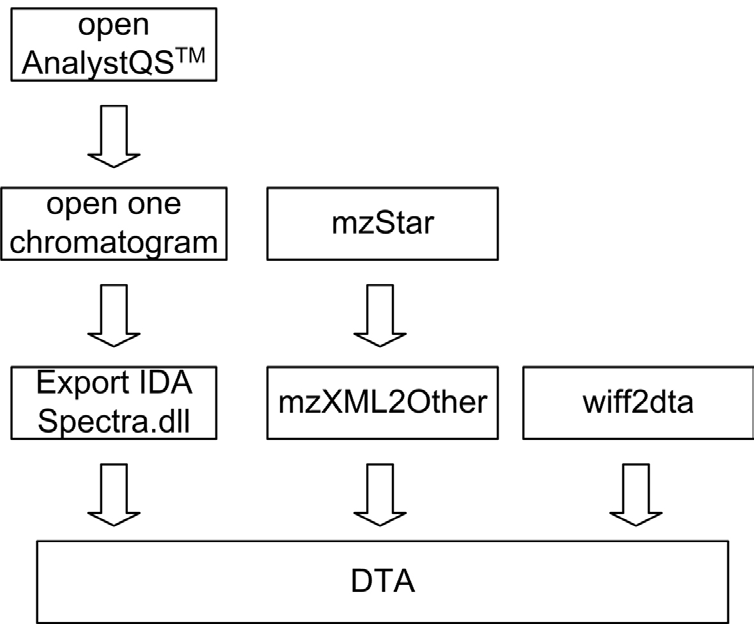
Schematic diagram of the workflows of the three conversion methods. For conversion of more than one wiff file, the whole process has to be repeated when using Export IDA Spectra.dll or mzStar, but not when using wiff2dta.

Applied Biosystems mass spectrometers are controlled by a software called Analyst™. This software is used for data evaluation purposes, too. It offers a possibility to integrate extensions called "scripts". One of these scripts available from the manufacturer [4] is "Export IDA Spectra.dll", the only known possibility besides the mzStar from SASHIMI Project [5] to export DTA files from Applied Biosystems ESI data. Using the tools provided by SASHIMI results in two steps: first mzStar must be used to create an XML [6] document (mzXML Schema) as intermediate step, then mzXML2Other must be applied for creating DTA or other formats from the mzXML document, and thus conversion consumes a lot of time and computational power. mzStar is not designed for batch processing nor for converting more than one wiff file in a single run. The Analyst™ script itself requires each chromatogram being opened in Analyst™ per conversion, resulting in a lot of user interaction for each single export. This leads to the effect that batch processing is impossible in both cases and only one binary file can be converted at once. A schematic diagram of the conversion method workflows is shown in figure 1.

Another script named mascot.dll provides support for invocating Mascot™ as protein identification algorithm using Applied Biosystems Analyst™. Such a script does not exist for Sequest™. In most proteomics labs support for Mascot™ as well as for Sequest™ is needed, because these two algorithms are most commonly used in this research field. Although the additional information that can be stored in mzXML is needed in the case of quantitative proteomics experiments based on isotopic labelling of peptides (ICAT [7] or SILAC [8]), this format can be read neither by Mascot™ nor by Sequest™.

We decided to develop a tool for converting data obtained from Applied Biosystems QStar™, providing features like batch processing in an operatorless high throughput environment. If no ER, NL or Prec scans are used, data acquired using a QTrap™ 2000/4000 can be converted, too. This tool is named wiff2dta.

## Implementation

The implementation was done according to the Analyst™ Cookbook, a documentation available from Applied Biosystems upon request. wiff2dta is implemented in Visual Basic™ (Microsoft Corp.) because ActiveX™ is provided as the one and only application programming interface (API) by Applied Biosystem's Analyst™ software. Therefore, this is needed for accessing the binary wiff files. Thus, this tool is operating system dependant and only runs on Windows™ (Microsoft Corp.) systems. We use the code provided by the Analyst™ software API in order to benefit from new releases and maintain coherence.

**Figure 2:**
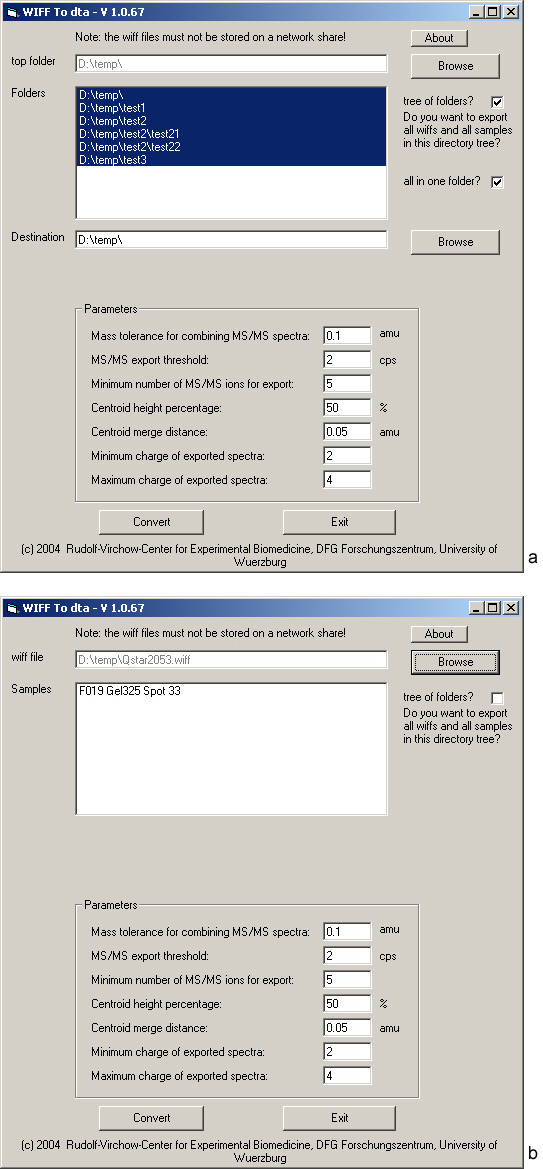
a) The graphical user interface of wiff2dta in directory-mode. This is the only form requiring user interaction. By klicking the button "About", a copyright message and the usage for batch-mode will be displayed. The usage is shown in figure 2. Clicking on the button "Convert" starts the conversion immediately. In directory mode, the tree of folders is listed and folders can be selected for being processed. b) The graphical user interface of wiff2dta in file-mode. The samples are listed and can be selected for conversion. The lower half of this form is identical for both the file- and the directory-mode

The program has two modes of user interaction: one provides a graphical user interface (GUI) and requires user interaction (GUI-mode); the other uses command-line parameters and suppresses the GUI as no user interaction is required (batch-mode). In batch mode, automation of conversion processes can be achieved. The GUI is shown in figure 2. Conversion can be done in two modes. On one hand only a single binary file can be selected for conversion (file-mode). On the other hand, a whole directory tree can be traversed and all binary ESI MS/MS files in all (or only selected) folders can be converted in one run (directory-mode). For example this mode can be used to convert a folder full of MS/MS data at once. In file-mode distinct samples of one data file can be marked for conversion, if desired. In directory-mode, each sample of each ESI MS/MS file is processed. Used in directory-mode, wiff2dta can be forced to save all resulting DTA files in one single folder by checking "all in one folder". Otherwise, the converted files are stored in a single folder with the name derived from the source ESI MS/MS data file. This folder is placed in the same directory where the corresponding binary file was found.

The conversion itself can be controlled by entering appropriate values in the text fields displayed under the title "Parameters", shown in figure 2. Parameters are "Mass tolerance for combining MS/MS spectra", "MS/MS export threshold", "Minimum number of MS/MS ions for export", "Centroid height percentage", "Centroid merge distance", "Minimum charge of exported spectra" and "Maximum charge of exported spectra". These are parameters of identical function as used by the export of DTA provided by Applied Biosystems' script. wiff2dta produces the same values as this tool, as shown in table 1. Support for other formats, like mascot generic format (MGF) [9] and mzXML [10] will be added. We first focussed on high throughput for conversion into DTA in order to be able of feeding our search programs efficiently.

**Figure 3:**
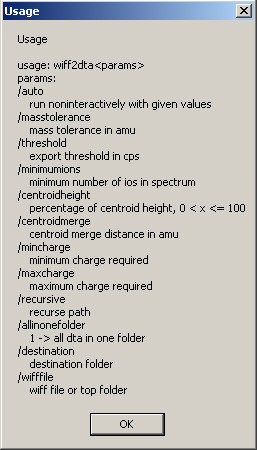
The parameters for batch-mode enabling wiff2dta being integrated in automated environments. All parameters can be controlled using the command line.

wiff2dta is able to be integrated in automation and high throughput environments. This can be achieved making use of the command line options. All parameters and modes can be controlled by command-line parameters. These are shown in figure 3. Every GUI parameter has a corresponding command line option. Batch-mode is entered by providing the parameter /auto at the command-line. If this is not present, the values provided override the defaults in the GUI and the form will be displayed.

## Results

The program can be started in multiple instances, resulting in parallel processing. Using this feature, it is possible to use several processors on one computer. Additional to this, wiff2dta is about 10% faster than the original tool provided by Applied Biosystems and about 20 times faster than mzStar of the Sashimi project. See table 2. During a 24 hour conversion, the 10% performance gain in savings of about 2.5 hours using the tool original tool provided by Applied Biosystems.

## Conclusions

wiff2dta demonstrates improvements in reducing computation time by exploiting a range of optimizations in coding and using the COM interfaces to Analyst™. Useful features like the capability of being integrated in batch processes and mass data processing lead to immense time savings, too.

## Availability and requirements

wiff2dta has to be installed in the BIN directory of an installed Analyst™ version 1.3 or higher. The installation consists just of copying the file wiff2dta.exe into this directory. If desired, a link to the program file can be created that can be placed onto the desktop or into the start menu.

The program is freely available from Applied Biosystems (UK) upon request and freely available via  and  for download.

## List of abbreviations used

API: application programming interface

DNA: desoxyribonuclein acid

DTA: file extension ms spectra data in Sequest™ format

ER: enhanced resolution

ESI: electron spray ionization

GUI: graphical user interface

MS: mass spectrometry, mass spectrometer

MGF: mascot generic format, file extension used for this format

NL: neutral loss

Prec: precursor ion

TOF: time-of-flight

WIFF: file extension of Applied Biosystems raw data files

## Authors' contributions

AB implemented the program and made a draft of the manuscript. RPG and AS contributed with ideas and proofread the manuscript. RPG supervised the final testing. All authors have read and approved the final manuscript.

## Figures and Tables

**Table 1:** Output in DTA format of the original DTA converter provided by the manufacturer (Export IDA Spectra.dll) and mzStar compared with the results of wiff2dta. All three used the same source file. The output of mzStar differs completely because this tool does not use any grouping of spectra as Export IDA Spectra and wiff2dta do. In DTA format, the first line is reserved for the mass of the parent ion and its charge. The other lines consist of pairs of m/z values and the corresponding intensities.

Export IDA Spectra		mzStar		wiff2dta	
1012.5922	2	1013	2	1012.5922	2
211.0680	4.0000	55.0534	3	211.0680	4.0000
221.1128	4.0000	56.0418	2	221.1128	4.0000
273.1110	7.0000	56.0451	2	273.1110	7.0000
274.0969	3.0000	56.0484	2	274.0969	3.0000
281.0214	4.0000	56.0518	2	281.0214	4.0000
281.0846	4.0000	60.0487	2	281.0846	4.0000
291.1109	4.0000	69.0642	4	291.1109	4.0000
294.1899	4.0000	69.0679	7	294.1899	4.0000
312.0073	2.0000	69.0716	9	312.0073	2.0000
493.2674	2.0000	69.0753	4	493.2674	2.0000
506.3299	2.0000	69.079	2	506.3299	2.0000
507.2403	2.0000	72.0658	3	507.2403	2.0000
507.3192	2.0000	72.0696	13	507.3192	2.0000
507.3693	2.0000	72.0734	17	507.3693	2.0000
508.2601	2.0000	72.0772	34	508.2601	2.0000

**Table 2:** Performance comparison of Export IDA Spectra, mzStar and wiff2dta on the same computer. wiff2dta is generally faster than the other tools.

File	Number of MS/MS spectra	mzStar	Export IDA Spectra	wiff2dta
Qstar0803	679	241.5 s	24.5 s	21 s
Qstar2053	992	426 s	28 s	24 s
Qstar2128	1652	1804 s	97 s	87.5 s
